# Effects of non-invasive spinal cord stimulation on lower urinary tract, bowel, and sexual functions in individuals with chronic motor-complete spinal cord injury: Protocol for a pilot clinical trial

**DOI:** 10.1371/journal.pone.0278425

**Published:** 2022-12-13

**Authors:** Soshi Samejima, Claire Shackleton, Laura McCracken, Raza N. Malik, Tiev Miller, Alex Kavanagh, Amandeep Ghuman, Stacy Elliott, Matthias Walter, Tom E. Nightingale, Michael J. Berger, Tania Lam, Rahul Sachdeva, Andrei V. Krassioukov

**Affiliations:** 1 Faculty of Medicine, International Collaboration on Repair Discoveries, University of British Columbia, Vancouver, BC, Canada; 2 Division of Physical Medicine and Rehabilitation, Department of Medicine, University of British Columbia, Vancouver, BC, Canada; 3 Department of Urologic Sciences, University of British Columbia, Vancouver, BC, Canada; 4 Department of Surgery, St. Paul’s Hospital, Vancouver, BC, Canada; 5 Department of Psychiatry, University of British Columbia, Vancouver, British Columbia, Canada; 6 Department of Urology, University Hospital Basel, University of Basel, Basel, Switzerland; 7 School of Sport, Exercise and Rehabilitation Sciences, University of Birmingham, Birmingham, United Kingdom; 8 Centre for Trauma Sciences Research, University of Birmingham, Edgbaston, Birmingham, United Kingdom; 9 GF Strong Rehabilitation Centre, Vancouver Coastal Health, Vancouver, BC, Canada; 10 School of Kinesiology, University of British Columbia, Vancouver, BC, Canada; PLoS ONE, UNITED STATES

## Abstract

**Introduction:**

Electrical spinal cord neuromodulation has emerged as a leading intervention for restoring autonomic functions, such as blood pressure, lower urinary tract (LUT), bowel, and sexual functions, following spinal cord injury (SCI). While a few preliminary studies have shown the potential effect of non-invasive transcutaneous spinal cord stimulation (tSCS) on autonomic recovery following SCI, the optimal stimulation parameters, as well as real-time and long-term functional benefits of tSCS are understudied. This trial entitled “Non-invasive Neuromodulation to Treat Bladder, Bowel, and Sexual Dysfunction following Spinal Cord Injury” is a pilot trial to examine the feasibility, dosage effect and safety of tSCS on pelvic organ function for future large-scale randomized controlled trials.

**Methods and analysis:**

Forty eligible participants with chronic cervical or upper thoracic motor-complete SCI will undergo stimulation mapping and assessment batteries to determine the real-time effect of tSCS on autonomic functions. Thereafter, participants will be randomly assigned to either moderate or intensive tSCS groups to test the dosage effect of long-term stimulation on autonomic parameters. Participants in each group will receive 60 minutes of tSCS per session either twice (moderate) or five (intensive) times per week, over a period of six weeks.

Outcome measures include: (a) changes in bladder capacity through urodynamic studies during real-time and after long-term tSCS, and (b) resting anorectal pressure determined via anorectal manometry during real-time tSCS. We also measure assessments of sexual function, neurological impairments, and health-related quality of life using validated questionnaires and semi-structured interviews.

**Ethics and dissemination:**

Ethical approval has been obtained (CREB H20-01163). All primary and secondary outcome data will be submitted to peer-reviewed journals and disseminated among the broader scientific community and stakeholders.

## Background

Individuals with spinal cord injury (SCI) experience a myriad of less visible but still debilitating autonomic dysfunctions, in addition to paralysis [1,2]. Restoration of autonomic functions, specifically lower urinary tract (LUT), bowel and sexual functions, are rated among the highest priorities for recovery in individuals with SCI [3,4]. Impairments in sexual, LUT, and bowel functions can lead to life-threatening cardiovascular consequences, such as extreme hypertensive episodes of autonomic dysreflexia, which are triggered by stimuli arising from these pelvic organs [5–9]. These dysfunctions affect all aspects of life following SCI and are critical determinants of dignity, autonomy, health, and survival among the affected individuals with SCI [4]. Despite this, until recently, the vast majority of research in individuals with SCI has focused on motor paralysis, whereas more devastating ‘invisible’ autonomic disabilities, remain relatively understudied [10,11].

Common impairments in sexual function following SCI include erectile and/or ejaculatory disorders in males, and reduced vaginal lubrication in females, with both sexes experiencing orgasmic difficulties or anorgasmia, as well as perceived deficits in sexual drive and sexual satisfaction [12,13]. Compromised orgasmic ability is reported in 75% and 43% of men and women with SCI, respectively [14,15]. Several studies have identified sexual function as one of the most important priorities for individuals with SCI [3,16,17].

The LUT has two functional components: the urinary bladder (detrusor) as well as the unit of the bladder neck, urethra, and urethral sphincter, which are responsible for low pressure continent storage of urine and periodic, self-determined release of stored urine (voiding) [18]. Impairment or loss of LUT control following SCI inevitably results in LUT dysfunction and consequently reduces health-related quality of life [19]. Uncontrolled intravesical pressure can jeopardize the entire urinary tract, leading to vesico-ureteral-renal reflux, urinary tract infections, bladder and renal stone formation, and impaired renal function in over 90% of individuals with neurogenic LUT dysfunction [20].

Neurogenic bowel dysfunction following SCI can present as constipation, impaired colonic motility, and loss of volitional control resulting in episodes of faecal incontinence [21]. Over 90% of individuals with SCI report at least one bowel issue, including prolonged time needed for bowel management, which can last up to two hours [22]. Over 60% of individuals with SCI report that bowel dysfunction adversely impacts health-related quality of life [23–25].

Pelvic organs share spinal cord circuitry and rely on a low-pressure system to store urine or transport stool. Bladder and bowel elimination requires an increase in intravesical and intraabdominal pressures, with simultaneous reduction of the outlet obstruction to a minimum, by relaxing their respective external sphincters. We have extensively demonstrated that life-threatening cardiovascular dysfunctions, such as autonomic dysreflexia, are commonly associated with stimuli arising from pelvic organs or iatrogenic procedures in individuals with SCI [6–9]. As a precautionary measure to ensure participant safety, procedures or interventions applied below the level of SCI should be evaluated for adverse cardiovascular events [26].

Electrical neuromodulation of the spinal cord has received significant attention in recent years as a promising approach to manage various sequelae of SCI [10,11,27]. The therapeutic potential of spinal cord stimulation (SCS) to activate disconnected neuronal circuits below the lesion is supported by several studies that demonstrate motor recovery [28–32]. Previous clinical studies have demonstrated the use of epidural stimulation for autonomic recovery of LUT [33,34], bowel [35,36] and sexual [29,37] functions. Though these findings are encouraging, a major drawback of epidural stimulation is the need to undergo highly invasive and expensive surgical procedures for electrode implantation [38,39]. Moreover, the inability to reposition implanted electrodes or increase the number of electrodes is a significant limitation of epidural stimulation. The amelioration of sexual, bowel and LUT dysfunctions likely requires changing electrode position to correspond with the spinal segments responsible for these specific organs. As an alternative, there is a growing body of evidence that supports the use of transcutaneous spinal cord stimulation (tSCS) as a non-invasive therapy, which allows electrode repositioning, to promote autonomic recovery in these pelvic organs [40–42].

## Trial objectives

We hypothesize that tSCS promotes recovery of LUT, bowel and sexual functions, without any adverse cardiovascular effects, in individuals with motor-complete SCI at T6 or above. To test this hypothesis, we plan to conduct a pilot single-blinded clinical trial of tSCS for autonomic dysfunctions with the following aims: to test the safety and feasibility of real-time tSCS in promoting recovery of LUT and bowel functions (AIM 1), and to assess the safety and dosage effect of long-term tSCS, on LUT, bowel, and sexual functions by comparing moderate (twice per week) versus intensive (five times per week) tSCS (AIM 2). The trial objectives and study design are developed in accordance with the CONSORT guideline for pilot trials [43,44].

## Methods and analysis

### Study design and setting

**Fig 1:**
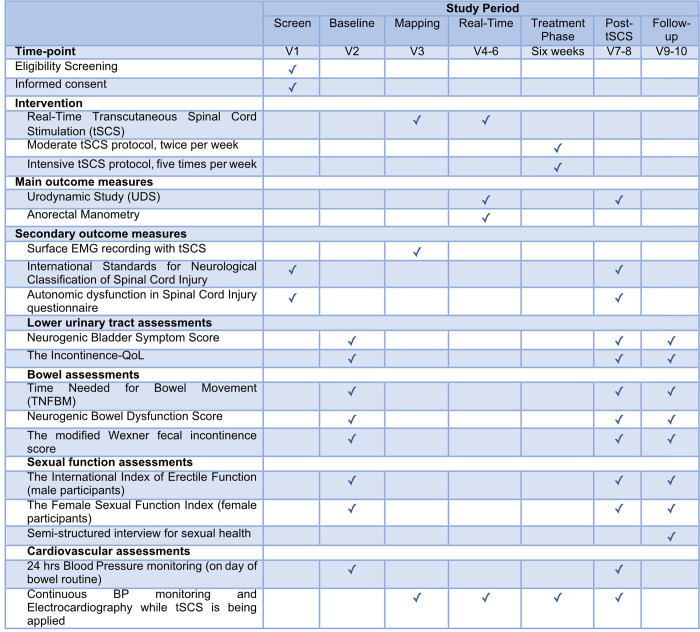
The SPIRIT schedule of enrolment, interventions, and assessments.

An overview of the experimental schedule, intervention and outcome is illustrated in Fig 1. The trial involves baseline assessments, electromyography (EMG) mapping, and assessments of the real-time and long-term effects of tSCS on sexual, LUT, and bowel dysfunctions in individuals with cervical or upper thoracic, at the sixth thoracic segment (T6) or above, motor-complete SCI. With the exception of anorectal manometry testing conducted at the Gastroenterology Clinic, St. Paul’s Hospital, Vancouver, Canada, all experimental procedures will be conducted at the International Collaboration on Repair Discoveries (ICORD), Blusson Spinal Cord Centre (BSCC), University of British Columbia (UBC), Vancouver, Canada. The interdisciplinary research team consists of physiatrists, urologists, colorectal surgeons, physical therapists and researchers.

**Fig 2:**
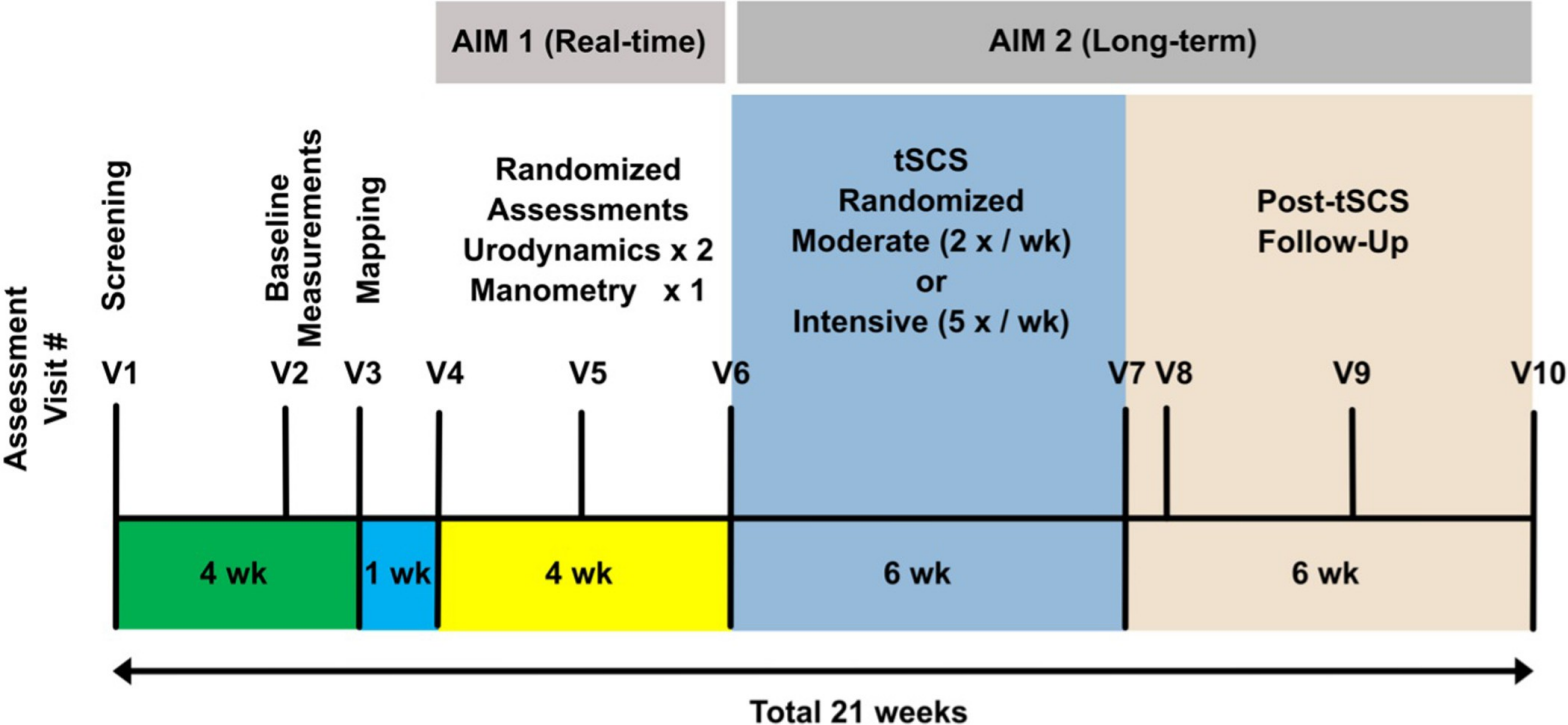
Trial design: Study timeline showing each study visit and the duration of the entire study. tSCS = Transcutaneous spinal cord stimulation, V = visit, and wk = week(s).

**Fig 3:**
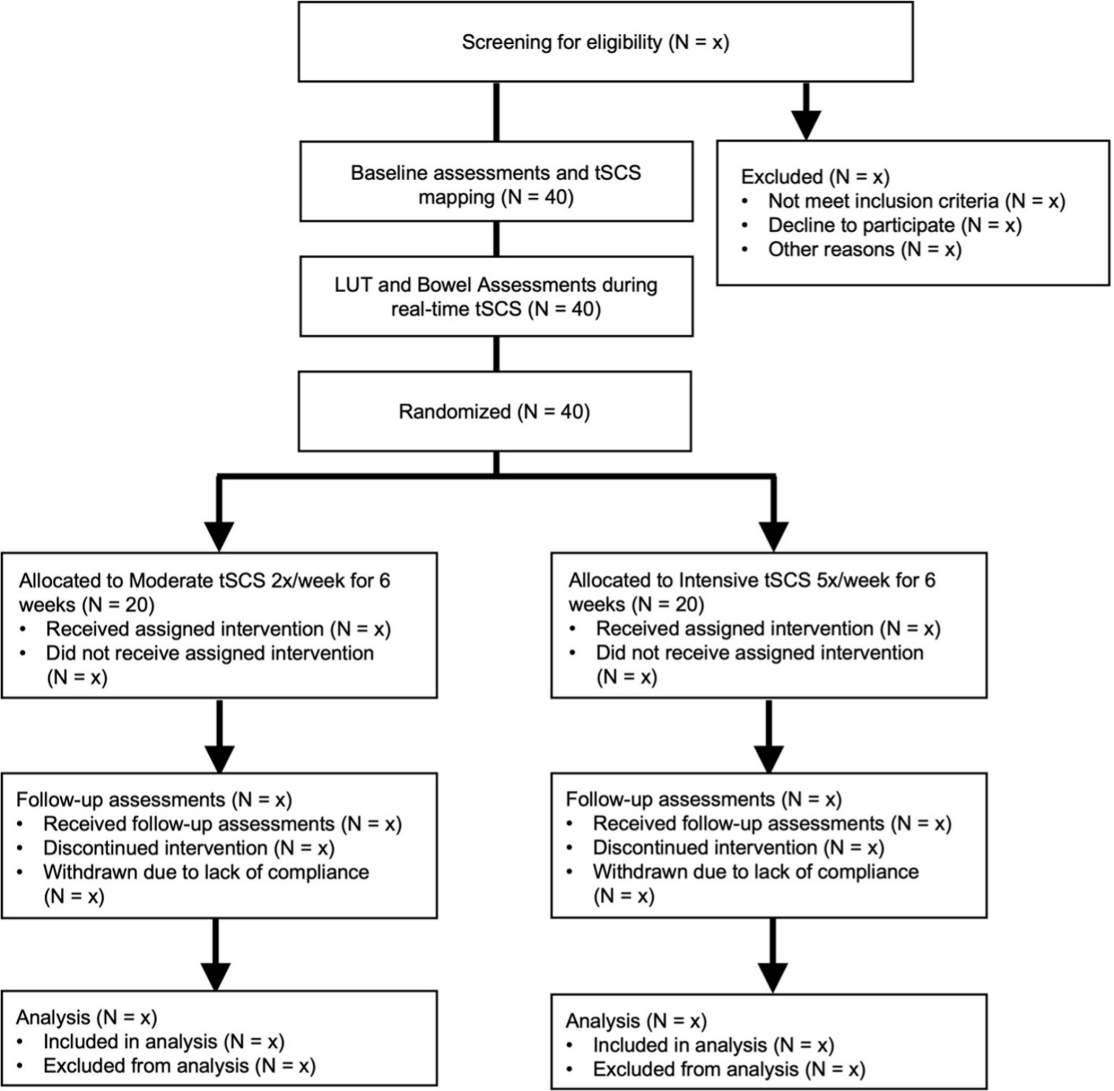
Trial flow chart. LUT = Lower Urinary Tract, tSCS = transcutaneous spinal cord stimulation, wk = week.

The experimental design and trial flow are illustrated in Figs 2 and 3, respectively. Following screening for the inclusion and exclusion criteria and informed consent (Visit 1), participants will undergo baseline evaluations for sexual, LUT, and bowel dysfunction severity using validated questionnaires. The International Standards for Neurological Classification of Spinal Cord Injury (ISNCSCI) examination will be performed by an experienced and trained investigator (AKV and MB; Visit 2). Spatiotemporal mapping of spinal cord segments will be performed with using surface EMG responses for target muscles involved in LUT and bowel control (Visit 3). Eligible participants will then undergo baseline quantitative LUT and bowel function measurements using a randomized counter-balanced approach. Participants will be allocated into two distinct pathways; (a) participants start with urodynamic studies (UDS) (Visits 4 and 5) and anorectal manometry (Visit 6), or conversely (b) participants will begin with anorectal manometry (Visit 4), followed by UDS (Visits 5 and 6). This approach will be used to avoid an order effect. Subsequently, individuals will be randomized to two long-term tSCS pathways; (a) moderate tSCS group, consisting of two sessions per week, or (b) intensive tSCS group, five sessions per week. Following six weeks of intervention, post-stimulation evaluations will be conducted (Visits 7 and 8), which involve UDS, ISNCSCI, and the use of validated questionnaires to assess perceived sexual, LUT, and bowel dysfunctions. Finally, participants will have two follow-up evaluations using the same questionnaires at three weeks and six weeks after the cessation of tSCS (Visits 9 and 10). Allocation will be open for the frequency of interventions. A statistician not involved in the trial will generate the randomization sequence using permuted blocks of varying size.

### Recruitment

Forty participants, which is justified based on the power calculation below, will be recruited. Potential participants will be identified through a variety of recruitment strategies including posters, social media advertising, letters of initial contact and upon request via email/ referral. Inclusion and exclusion criteria are outlined in Table 1.

**Table 1:** Participant inclusion and exclusion criteria.

Inclusion criteria	Exclusion criteria
1. Resident of British Columbia, Canada with active provincial medical services plan 2. 18–65 years of age 3. Chronic traumatic SCI at or above the T6 spinal segment 4. >1-year post injury, at least 6 months from any spinal surgery 5. Documented presence of LUT dysfunction 6. Documented presence of bowel or sexual dysfunction 7. American Spinal Injury Association Impairment Scale Grade A or B 8. Greater than or equal to antigravity strength in deltoids and biceps bilaterally 9. Hand function sufficient to perform clean intermittent catheterization (CIC) or a committed caregiver to provide CIC for management of urinary bladder drainage 10. Willing and able to comply with all clinic visits and study-related procedures 11. Able to understand and complete study-related questionnaires 12. No painful musculoskeletal dysfunction, unhealed fracture, pressure sore, or active infection that may interfere with testing activities 13. Medication dosage must be stable for period of 4 weeks prior to participation 14. Women of childbearing potential must not intend to become pregnant, or be currently pregnant, or lactating	1. Presence of severe acute medical issues that in the investigator’s judgement would adversely affect the participant’s ability to participate in the study. Examples include but are not limited to clinically significant renal or hepatic disease; acute urinary tract infections; pressure sores; active heterotopic ossification; newly changed antidepressant medications [tricyclics]; or unstable diabetes. 2. Recent treatment with OnabotulinumtoxinA into the detrusor muscle (within 6 months of the baseline visit) 3. Ventilator dependent 4. Clinically significant depression or ongoing drug abuse 5. Any implanted metal in the skull or presence of pacemakers, implantable defibrillators, neurostimulators, cochlear implants, or drug delivery pumps in the trunk 6. Oral baclofen dose greater than 60mg 7. Cardiovascular, respiratory, bladder, or renal disease unrelated to SCI or presence of hydronephrosis or presence of obstructive renal stones 8. Any implanted metal in trunk or spinal cord under the electrode application sites 9. Severe anemia (hemoglobin <8 g/dl) or hypovolemia 10. Participant is a member of the investigational team or his /her immediate family 11. Participant has undergone electrode implantation surgery 12. Participant has swollen, infected, and inflamed areas or open wounds on the area of stimulation

### Blinding

As this is an open-label trial, neither the participants nor the researchers will be blinded. However, outcome assessors will be blinded.

### Interventions

We will use an isolated spinal cord stimulator (TESCoN, SpineX Inc., USA) for non-invasive tSCS [41], that has been approved by Health Canada as a class II medical device for use in this clinical trial (Application# 336738, approved on March 25, 2022). The waveform of the device comprises a high carrier frequency (10kHz) overlapping a regular 1ms pulse with a low burst frequency (1-50Hz) component. The stimulator delivers electrical current through self-adhesive round cathode electrodes (1.25-inch diameter), which will be placed on the skin between spinous processes, at the midline over the vertebral column. Two rectangular 2- x 3.5-inch self-adhesive anode electrodes will be placed bilaterally on the skin overlying the iliac crests. tSCS will be delivered individually between spinous processes from T10-L2 vertebrae based on the results of the mapping study described below. Stimulation parameters will be optimized based on skeletal muscle EMG responses when tSCS is applied at various frequencies (1-50Hz) and current amplitudes (1-130mA) using a previously described protocol [45–47]. A skin temperature probe (MLT422, AD Instruments, USA) will be positioned under cathode as a safety precaution. The most effective stimulation parameters to target spinal segments responsible for LUT and bowel control will be established and utilized for determining the real-time (AIM 1) and long-term effects of tSCS (AIM 2). For the long-term trial, participants will receive 60 minutes of optimized tSCS in a seated position two sessions per week (moderate dose group, n = 20) or five sessions per week (intensive dose group, n = 20), for six weeks.

### Assessments

Following screening and the provision of informed consent, participants will be enrolled in the trial. Specific outcome measures for each aim at each timepoint are listed in Fig 1. Continuous blood pressure monitoring will be used as a safety measure to detect potential adverse cardiovascular events throughout the trials in accordance with the Standard Protocol Items: Recommendations for Interventional Trials (SPIRIT) guidelines [48]. The executive committee consisting of the trial principal investigator, researchers, and research coordinators will convene regularly to discuss any logistical issues.

We will conduct individualized tSCS mapping in order to elicit optimal skeletal muscle activation corresponding to specific spinal cord segments involved in LUT and bowel control. Identifying specific stimulation parameters and electrode placement are the secondary outcomes. The effect of real-time tSCS on LUT and bowel functions will be evaluated during tSCS. Bladder capacity assessed via UDS and resting anorectal sphincter pressure assessed via anorectal manometry are outcomes for LUT and bowel functions, respectively. Secondary outcomes will include the perceived desire to void, the presence of detrusor overactivity, squeezing anorectal sphincter pressure, recto-anal inhibitory reflex, anorectal sensation via anorectal manometry. Time needed for bowel management with tSCS will be reported as a secondary bowel function outcome measure.

The potential efficacy of long-term tSCS for LUT and bowel functions will be evaluated before and after six weeks of tSCS. We will collect bladder capacity and resting anorectal sphincter pressure as outcomes for LUT and bowel functions, respectively. As secondary outcomes, we will assess the impact of tSCS on LUT- and bowel-related daily activities and quality of life through validated questionnaires including the Neurogenic Bladder Symptom Score (NBSS), the Incontinence-quality of life (incontinence-QoL), the Neurogenic Bowel Dysfunction Score (NBDS), the modified Wexner fecal incontinence (WFI) score and time needed for bowel management. In addition, we will use biological sex-specific validated sexual function questionnaires, including the Female Sexual Function Index (FSFI) for females and the International Index of Erectile Function (IIEF-15) for males. The aforementioned questionnaires also will also be given at the follow-up (Visits 9 and 10) to assess the sustained effects of tSCS on these autonomic functions. Upon completion of the study, we will conduct a semi-structured interview to gather additional information on sexual health-related quality of life. The ISNCSCI and Autonomic Dysfunction following Spinal Cord Injury (ADFSCI) questionnaires will be used before and after the long-term tSCS to assess the degree of neurological impairment and general autonomic dysfunction. We will also record recruitment rate, retention rate and details of adverse events as feasibility measures of this trial.

### Outcomes

UDS along with surface pelvic floor muscle EMG (Aquarius-TT, Laborie Model 94-R03-BT, Canada),will be performed (AIM 1) in accordance with Good Urodynamic Practices recommended by the International Continence Society (ICS) [49]. First, baseline UDS will provide pivotal information on the individual’s LUT function, including the presence of detrusor overactivity, bladder compliance, maximum detrusor pressure, the perception of the desire to void, voided volume in case of voluntary voiding or leaked volume in case of detrusor overactivity incontinence, post-void residual volume, presence of detrusor sphincter dyssynergia (DSD) and amplitude of pelvic floor muscle EMG activation. Subsequently, we will start tSCS with pre-determined parameters from Aim 1 and then repeat filling of the bladder to assess the efficacy of real-time tSCS on storage function, e.g. cystometric capacity and detrusor pressure (AIM 1). In the second UDS visit, UDS is performed in conjunction with tSCS using the same stimulation parameters. This is done to evaluate the reproducibility of improved storage function to control the order effect (first filling vs. second filling in the same UDS visit) and between different sessions. In the second bladder filling of the second UDS visit, we refill 60–75% of bladder capacity without tSCS and then start tSCS at 1Hz with predetermined intensity to assess voiding response to tSCS. A final UDS will be conducted to assess the long-term effects of tSCS after six weeks of treatment (AIM 2). Our pilot data demonstrated the utility of epidural stimulation in modulating detrusor pressure and external anal sphincter/pelvic floor muscle tone during UDS [36]. Sacral neuromodulation also resulted in ≥50% improvement in LUT function compared to baseline [50].

Anorectal manometry is a well-established methodology that provides a direct assessment of anorectal sphincter pressure and anorectal coordination during simulated defecation. The assessment is widely accepted, easy to perform and well-tolerated among participants [51]. This assessment is performed by a colorectal surgeon using high-resolution anorectal 3D probe with 16 axial x 16 circumferential sensors and a balloon tipped solid-state catheter (Manoscan, Medtronic, USA). In the anorectal manometry visit, we will first conduct a standard of care assessment [52]. Resting and squeezing sphincter pressure will be determined from measurements of sphincter pressure from the anal verge to the distal rectum. Recto-anal inhibitory reflex, first sensation, urge and maximum tolerated volume are also recorded. Subsequently, we will repeat the same assessment with real-time tSCS using predetermined parameters. Previous evidence shows that sacral neuromodulation resulted in ≥50% improvement in resting (continence) and squeezing (defaecation) sphincter pressure compared to baseline [53].

### Secondary outcomes

#### Stimulation mapping and neurological function

Surface EMG will be conducted (Delsys, USA) as per an established protocol to record muscle activation during mapping with and without tSCS [54,55]. We will elicit spinal motor evoked potentials concurrent tSCS across potential motor targets: T10-T12 = lower abdominal (rectus abdominis), L1-L2 = hip flexor (rectus femoris), L3 = knee extensors (vastus medialis/lateralis), L4 = ankle dorsiflexors (tibialis anterior), S1 = ankle plantar flexors (gastrocnemius/soleus), S2-S3 = knee flexors (biceps femoris), and S2-S4 = pelvic floor muscles. The recruitment curves of peak-to-peak responses to tSCS will be analysed to identify optimal, target-specific stimulation parameters. The site for each EMG sensor will be prepared by cleaning the skin with rubbing alcohol and removing any hair with a razor. The EMG sensors will be affixed to the skin over a given area using medical-grade adhesives. Data will be sampled at 2000Hz, filtered with bandpass filter of 30Hz– 500Hz, and stored for offline analysis. The recruitment (stimulus-response) curves of peak-to-peak responses to tSCS will be analysed to identify optimal, target-specific stimulation parameters. Based on the motor threshold and maximum response of spinal motor-evoked potentials, the optimal electrode placement and parameters will be determined.

The ISNCSCI will be administered at baseline, after six weeks of tSCS and at study completion. All key muscles, light touch and pinprick sensation of the upper limb, lower limb and trunk will be assessed. In accordance with the 2019 revision, ISNCSCI examinations will assess both motor and sensory neuropathology [56]. The composite score will be used to measure changes in the neurological level of injury and American Spinal Injury Association Impairment Scale (AIS) grade. The ISNCSCI is designated as a common core data element according to the National Institute of Neurological Disorders and Stroke recommendations [57].

The ADFSCI questionnaire is comprised of 24 self-reported items covering four domains, including general, medications, autonomic dysreflexia, and hypotension symptoms and severity [58]. Ten and seven items are specific to autonomic dysreflexia and hypotension, respectively, and are scored on a 5-point scale. The scale will assess the frequency and severity of hypertensive and hypotensive symptoms under various circumstances.

#### Lower urinary tract function

The NBSS, comprises of 23 questions covering three domains, including incontinence, storage & voiding, and specific consequences, as well as one question on quality of life [59].

The Incontinence-QoL (I-QoL) comprises ten questions covering three domains, including avoidance and limiting behaviour, psychosocial impacts, and social embarrassment, which are summarised as a total score. All scores, for each domain and a total, will be transformed into a continuous scale value [60]. Both of these measures will provide a clinically meaningful outcome and have demonstrated both good validity and reliability.

#### Bowel function

Time needed for bowel management during conventional routines will be also measured on non-consecutive days (i.e., separated by 1–2 days between attempts). To compare time needed for bowel management, we will record the time from ‘suppository insertion’ to ‘when bowel evacuation is completed’ at the participant’s home. Pre- and post-intervention bowel management times will be compared to determine the long-term effect of tSCS on bowel function. Our recent case study shows that epidural stimulation was able to reduce bowel management time by ~55% [36].

Faecal continence severity will be assessed using the modified Wexner incontinence scale [61]. The scale includes items that take into account type, frequency and extent to which faecal incontinence alters an individual’s life, use of anti-diarrhoea medications and the ability to defer defaecation for 15 minutes. The NBDS questionnaire will be administered to evaluate the magnitude of bowel dysfunction. This measure has been shown to be clinically meaningful with good reproducibility and validity in SCI population [62]. This questionnaire comprises ten questions focusing on defaecation including frequency, duration, and clinical symptoms, constipation including use of aiding medication and digital stimulation, faecal incontinence including frequency, aiding medication, and flatus, and peri-anal skin problems. The consequential NBDS relates to four different neurogenic bowel dysfunction severity levels. In addition to the NBDS, the questionnaire assesses general participant satisfaction regarding current bowel function.

#### Sexual function

Self-reported magnitude of sexual dysfunction will be assessed using sex-specific questionnaires: The International Index of Erectile Function (IIEF-15) comprises 15 questions covering five domains, including erectile function, orgasmic function, intercourse satisfaction, and overall satisfaction [63]. The Female Sexual Function Index (FSFI) is comprised of 19 questions covering six domains, including desire, arousal, lubrication, orgasm, satisfaction and pain [64]. The aforementioned measures have been shown to be clinically meaningful with good reproducibility and validity [65,66]. Item 13 of the IIEF-15 and item 16 of the FSFI are used to assess overall sexual life satisfaction for males and females, respectively. A sexual health clinician, with experience providing clinical care to individuals with SCI, will conducts a semi-structured qualitative interview at trial completion (SE).

#### Cardiovascular monitoring

Cardiovascular monitoring will be used as a safety measure to detect and record potential adverse cardiovascular events, such as autonomic dysreflexia, during tSCS delivery and outcome assessments (AIM 1). BP and heart rate (HR) will be measured continuously (beat-by-beat) via finger photoplethysmography and electrocardiogram (Finapres NOVA, Finapres Medical Systems, Netherlands) corrected to brachial pressure (Dynamap Pro, GE Healthcare, USA). An attending physician will confirm episodes of autonomic dysreflexia, which are defined according to the international standards to document remaining autonomic function after SCI as an increase in systolic BP ≥20mmHg from baseline [67]. Autonomic dysreflexia will be managed with the implementation of an established clinical management protocol in accordance with the consortium of spinal cord medicine clinical practice guidelines [68]. Furthermore, during bowel routine, we will assess BP instability using a 24-Hr ambulatory Meditech Card(X)plore BP monitoring device (Meditech Card(X)plore, Meditech Ltd., Hungary), and a well-established clinical protocol [69].

### Statistical analysis

To determine the real-time effects of tSCS on LUT and bowel functions, all outcomes in AIM 1 will be assessed using a non-parametric one-way repeated measures analysis of variance (ANOVA) (i.e., Kruskal-Wallis test). Long-term effects of tSCS on voiding efficiency and resting anorectal pressure without tSCS will be assessed using a non-parametric two-way repeated measures ANOVA (at baseline and the completion of tSCS) to compare changes between moderate and intensive tSCS (AIM 2). Differences in continuous variables for questionnaire data (NBSS, NBD Score, Incontinence-QoL, IIEF-15 or FSFI) will be assessed using two-way repeated measures ANOVA. Differences in UDS, anorectal manometry, and faecal continence severity (baseline and the completion of long-term tSCS) will be assessed using Wilcoxon signed-rank tests. All analyses will be conducted using SPSS software (IBM Corp. Armonk, NY, USA) at an alpha (α) threshold of p ≤ 0.05 with Bonferroni correction. A complete thematic analysis is performed using contextual qualitative data collected from the sexual health interview. All missing data will be reported with an intention to treat analysis.

### Power calculation and sample size

A sample size calculation was performed using G*power software (version 3.0.10, Heinrich Heine Universität Dusseldorf, Germany) with power = 0.8 and α = 0.05. Data from participants that match our inclusion criteria (n = 5, level of lesion C5 –T10, AIS A or B) were used from a recent tSCS study [47] A minimum sample of 26 participants is required to detect a significant change in voiding efficiency [voided volume / (voided volume + post-void residual volume)] with real-time tSCS (stimulation off = 37.8 ± 40.6% vs. stimulation on = 58.2 ± 39.4%, effect size = 0.510). Presently, there are no long-term tSCS data with LUT and bowel outcomes that are consistent with those proposed in this protocol to facilitate a meaningful power calculation. Therefore, given this is a pilot study, and to account for possible attrition during long-term tSCS, we aim to investigate 40 individuals with SCI.

## Discussion and limitations

This trial is focused on patient-centered outcomes that are rated as priorities for recovery by individuals with SCI. The diversity of autonomic outcomes measured in this pilot trial will provide a comprehensive/robust assessment of autonomic dysfunctions and provide insight to enhance outcome selection for future definitive trials. The techniques employed to mediate autonomic dysfunctions and measure changes in various functions are well-validated for individuals with SCI and will be performed by a collaborative, multidisciplinary team of experienced clinicians and researchers. Meanwhile, the trial limitations include the lack of a sham stimulation group to control for any potential placebo effects and the lack of participant and investigator blinding to the intervention received by participants, although outcome assessors will be blinded.

## Data management and safety

All data collected (electronic or hardcopy documents) will be coded with unique identification numbers and stored centrally on the Empower database, a password-protected computer, or in a locked filing cabinet in a secure laboratory space only accessible to the study investigators. To ensure information quality and accuracy after publication, all data will be stored for 25 years, and then subsequently destroyed.

Any adverse events reported during the intervention will be documented with information pertaining to their severity and anatomical location. The study coordinator will immediately report adverse events to the ethics board and Data and Safety Monitoring Committee (DSMC), which is comprised of three external, independent physician scientists with no involvement in the study, as well as the appropriate ethics board. The DSMC is responsible for safeguarding the interests of trial participants, assessing the safety and efficacy of the interventions during the trial, and monitoring the overall conduct of the clinical trial. The DSMC also provides recommendations for continuing or discontinuing the trial and outcome data use for participants who discontinue the trial. The trial progress will be biannually reported to the DSMC.

## Ethics and dissemination

This pilot study will be conducted in accordance with the Declaration of Helsinki and is consistent with the International Conference on Harmonisation Good Clinical Practice Guidelines, as well as applicable regulatory requirements. The UBC Clinical Research Ethics Board (UBC CREB H20-01163, NCT04604951) has already approved this study protocol. The UBC CREB and DSMC will be contacted when the trial makes any important protocol modifications. The feedback base on functional outcome measure results can be provided to participants for their adherence and benefits. The results of this pilot trial will be presented at national and international conferences and will be published in peer-reviewed journals. All subsequent manuscripts will be reported in conjunction with the Consolidated Standards of Reporting Trials [43]. Additionally, a summary of the trial findings will be posted on the ICORD website and in magazines published by service organizations for people with SCI in provinces where participants were recruited (i.e., SCI BC). Extensive, individualized feedback will be provided to each participant upon completing the trial.

## Supporting information

S1 Checklist SPIRIT checklist and approved ethics protocol are included as a supplemental document. (DOC)

S1 File (PDF)
