# Supplementary material for: Effects of non-invasive spinal cord stimulation on lower urinary tract, bowel, and sexual functions in individuals with chronic motor-complete spinal cord injury: Protocol for a pilot clinical trial
Source: PLoS One. 2022 Dec 13;17(12):e0278425. doi: 10.1371/journal.pone.0278425 (PMC9746997; doi:10.1371/journal.pone.0278425)
Supplement: S1 File — (PDF) [file pone.0278425.s002.pdf]

**PROTOCOL TITLE**	***Below the belt: non-invasive neuromodulation to treat bladder, bowel and sexual dysfunction following spinal cord injury***
**PROTOCOL NUMBER**	H20-01163

List of abbreviations

ACVE	Adverse Cardiovascular Events
AE	Adverse Event
ABPM	Ambulatory Blood Pressure Monitor
AIS	American Spinal Injury Association Impairment Scale
ARM	Anorectal Manometry
ADFSCI	Autonomic Dysfunctions following SCI
AD	Autonomic Dysreflexia
BP	Blood Pressure
BSCC	Blusson Spinal Cord Centre
CIC	Clean Intermittent Catheterization
DSD	Detrusor Sphincter Dyssynergia
EMG	Electromyography
EUS	External Urethral Sphincter
FSFI	Female Sexual Function Index
GCP	Good Clinical Practice
GI	Gastrointestinal
HR-QoL	Health-Related Quality of Life
HR	Heart Rate
I-QoL	Incontinence-QoL
ICF	Informed Consent Form
ICORD	International Collaboration on Repair Discoveries
ICH	International Council for Harmonisation
IIEF-15	International Index of Erectile Function
LUT	Lower Urinary Tract
NBSS	Neurogenic Bladder Symptom Score
NBD Score	Neurogenic Bowel Dysfunction Score
NDO	Neurogenic Detrusor Overactivity
PHQ-9	Patient Health Questionnaire
QoL	Quality of Life
SCI	Spinal Cord Injury
TNFBM	Time needed for Bowel Movement
TCSCS	Transcutaneous Spinal Cord Stimulation
TEAE	Treatment Emergent Adverse Events
UDS	Urodynamics
WIS	Wexner Incontinence Score

Background literature review

In addition to devastating paralysis, individuals with SCI suffer from a myriad of less visible autonomic dysfunctions.[1,2] Restoration of autonomic functions, specifically lower urinary tract (LUT), bowel and sexual functions, are rated among the highest priorities for recovery in individuals with SCI.[3,4] Multiple studies have highlighted the staggering discrepancies between the priorities of people with SCI and the focus of research efforts to date.[3,5] Despite this, the vast majority of research is focused on overt disabilities following SCI (e.g. motor paralysis), whereas more devastating 'invisible' disabilities such as LUT, bowel and sexual dysfunctions, which are of higher priority than walking, are unfortunately understudied.

SCI disrupts crucial crosstalk between the spinal autonomic nervous system and supraspinal control centres. Therefore, contrary to the general perception, SCI results in not only motor paralysis but also in a pernicious cascade of severe impairments in vital autonomic functions such as LUT, bowel and sexual control.[1] Moreover, impairments in these functions lead to life-threatening cardiovascular consequences e.g. extreme hypertensive episodes of autonomic dysreflexia.[6–9] These dysfunctions affect all aspects of life post-SCI and are the most important determinants of dignity, autonomy, health and survival of the affected individual.

Bladder function [clinically referred to as LUT function]: The human LUT comprises two functional components: the urinary bladder (*detrusor*) as well as the unit of the bladder neck, urethra, and urethral sphincter, which are responsible for low pressure continent storage of urine and periodical, self-determined, complete release of the stored urine (*voiding*).[10] To properly execute both functional components, the LUT relies on an intact nervous system with coordinated control by cortical and spinal (thoracolumbar and sacral) centres.[10,11] Impairment or the loss of LUT control following SCI inevitably results in LUT dysfunction and consequently reduces HRQOL. Failure in urine storage (due to involuntary detrusor contraction and inability to voluntary activate the external urethral sphincter (EUS) and voiding (due to the inability to voluntary initiate) combined with detrusor-sphincter-dyssynergia DSD, a condition where the detrusor and the external urethral sphincter contract at the same time, results in high intravesical pressure and inefficient voiding with a significant post-void residual volume.[12] This pathologic, high-pressure situation jeopardizes the entire urinary tract long-term as it leads to vesico-ureteral-renal reflux, urinary tract infections, bladder stone formation, and impaired renal function – ultimately leading to significant disease burden and poorer HRQOL.[3] The fundamental cause underlying impairment of LUT function post SCI is loss of supraspinal control and disruption of crosstalk between the somatic and autonomic nervous systems.[1] The resulting condition, termed neurogenic LUT dysfunction, impacts over 90% of individuals with SCI.[13]

Bowel function is also significantly compromised after SCI, presenting as constipation, impaired colonic motility and loss of volitional control resulting in episodes of faecal incontinence - predisposing an individual to increased risk of long-term complications.[14] These impairments, coupled with impaired mobility and hand dexterity make bowel management a major life-altering problem.[15] Normal bowel function, i.e. focusing on the lower gastrointestinal (GI) tract, is regulated by a fine balance between somatic and autonomic (sympathetic, parasympathetic and enteric) control.[16] Moreover, voluntary defaecation relies on supraspinal control.[1] Despite the intricate crosstalk between multiple layers of neural control governing bowel function, it is the somatic and autonomic components of the central nervous system that regulate peristalsis/defaecation (i.e. another major concern in individuals with SCI). In most cases of SCI (i.e. cervical or high-thoracic),[17] the proximal colon has intact parasympathetic but not sympathetic supraspinal control, while the distal colon loses supraspinal autonomic control altogether.[1] Over 98% of individuals with SCI report at least one bowel issue e.g. Time needed for bowel

movement (TNFBM) lasting up to two hours [18] and over 60% report that bowel dysfunction adversely impacts HRQOL.[19–21]

The LUT and bowel share spinal circuitry and rely on a low-pressure system to store urine or arrange stool transportation. For elimination (i.e. voiding and defaecation), the LUT (*bladder*) and bowel (*rectum*) increase organ-specific, intravesical and intraabdominal pressures respectively, while reducing the outlet obstruction to a minimum, i.e. by relaxing their respective external (*urethral and anal*) sphincters. As with the LUT, continuous elevated pressure in the lower gastrointestinal (GI) tract places affected individuals at increased risk for haemorrhoids, anal fissures, diverticular disease, rectal bleeding, and rectal prolapse (a condition in which the rectal wall pushes out through the anus). Therefore, it is paramount to restore LUT and bowel function with respect to the intravesical and abdominal pressure (i.e. low during storage and appropriate increase during elimination). Thus, in order to assess LUT and bowel function, this proposal will utilize well-established and robust procedures i.e. urodynamics (UDS), along with external anal sphincter/pelvic floor surface electromyography (EAS/PF) for LUT function, and Anorectal Manometry (ARM) for lower GI function.

Severe impairments in sexual function following SCI include erectile dysfunction, ejaculatory disorders/ anejaculation in men and reduced vaginal lubrication in women, with both sexes experiencing orgasmic difficulties or anorgasmia as well as alterations in sexual drive and sexual satisfaction.[22] Obtaining and maintaining an erection that is sufficient for satisfactory sexual performance and to achieve ejaculation and orgasm (for both pleasure and biological fatherhood) is therefore a major concern for men following SCI, with up to 89% experiencing erectile difficulties.[3,22,23] Both men and women with SCI also experience compromised orgasmic ability, with a 75% and 43% incidence, respectively.[23,24] Several systematic reviews of the literature identified sexual function as one of the most important priorities for individuals with SCI[3,25,26] and improvements in sexual function is considered an instrumental aspect of successful rehabilitation.[27] The World Health Organization recognizes sexual function as a fundamental right and targeted approaches that may provide improvements in these outcomes are of utmost priority for individuals with SCI.

Finally, it is important to recognize that life-threatening cardiovascular dysfunctions post-SCI are commonly associated with these autonomic dysfunctions. In fact, Dr. Krassioukov's laboratory has extensively demonstrated that severe hypertensive episodes of autonomic dysreflexia are frequently triggered by stimuli arising from LUT, bowel or sexual organs.[7–9,28] Therefore, any intervention or stimulation applied below the level of SCI should also be evaluated for cardiovascular safety, as triggered autonomic dysreflexia could be associated with significant, life-altering ACVEs.[29]

This study will use the TEScON device is a non-invasive electrical device provides transcutaneous spinal cord stimulation for the treatment for patients with cardiovascular dysfunction, neurogenic lower urinary tract dysfunction and neurogenic bowel dysfunction, sexual dysfunction and sensorimotor impairments following spinal cord injury. Transcutaneous stimulation is applied to the spinal cord by electrodes connected to a battery-powered source which activates the nerves associated with autonomic function.

Purpose

The purpose of this project is to assess the safety and efficacy of non-invasive transcutaneous spinal cord stimulation (TCSCS) in promoting recovery of LUT, bowel and sexual function in individuals with SCI.

Objectives, Hypotheses and Outcomes

Objective 1: To establish specific spatiotemporal activation maps for TCSCS at various spinal cord segments known to be involved in LUT and bowel control.
Hypothesis 1: In light of recent findings on the efficacy of spinal cord stimulation to improve bowel and LUT function, the optimal setting for motor responses (i.e. mapping) will be revealed during TCSCS.
Primary outcome 1: An individualized TCSCS map (i.e. location and stimulation parameters) to elicit optimal skeletal muscle activation corresponding to spinal cord segments involved in LUT and bowel control

Objective 2: To test whether targeted short-term TCSCS is safe with respect to cardiovascular consequences (e.g. autonomic dysreflexia) and can repeatedly improve LUT and bowel functions in humans with chronic SCI.

Objective 2A) LUT function (safety/efficacy): To test whether targeted short-term TCSCS is safe and can induce detrusor contractions (*voiding function*) while relaxing the EUS, i.e. to avoid DSD and independently enhance EUS tone (*storage function*).
Hypothesis 2A): In light of recent findings demonstrating TCSCS improves LUT function, we hypothesize that short-term TCSCS will improve voiding and storage functions without ACVEs.
Outcome 2A): Changes from baseline in the following UDS parameters for bladder capacity/compliance:

i. Volume at the first sensation
ii. Intravesical pressure (Pves) at the first sensation
iii. Volume at the leakage point
iv. Pressure at the leakage point
v. Maximum bladder volume
vi. Pves at the maximum bladder volume

Objective 2B) LUT function (repeatability of TCSCS to improve UDS parameters): To test whether targeted short-term TCSCS will repeatedly improve UDS parameters across different stimulation sessions.
Hypothesis 2B): TCSCS will elicit reproducible improvements in UDS parameters without ACVEs.
<u>Outcome 2B)</u><u>:</u> Reproducibility of changes within urodynamic parameters during stimulation will be assessed during two consecutive urodynamic procedures. UDS parameters for bladder capacity/compliance:

i. Volume at the first sensation
ii. Pves at the first sensation

Objective 2C) Bowel function (safety/efficacy): To test whether targeted short-term TCSCS is safe and can result in an immediate increase in anorectal pressure.
Hypothesis 2C): TCSCS will increase anorectal pressure without adverse cardiovascular effects.
Outcome 2C*<u>)</u>*<u>:</u> Mean increase in maximum resting pressure by at least 50%.

Objective 3) LUT, bowel and sexual functions (long-term TCSCS): To test whether targeted long-term TCSCS (6-week trial) is safe and can improve urinary and faecal continence in humans with chronic SCI.

Objective 3A) To test whether targeted long-term TCSCS can improve urinary and faecal continence.

Hypothesis 3A) Long-term TCSCS will improve urinary and faecal continence. These improvements will be greater in individuals who complete intensive long term TCSCS compared to individuals who complete moderate long term TCSCS.
Outcome 3A): Mean decrease in the frequency of urinary and faecal incontinence by at least 50%.

Objective 3B) To test whether targeted long-term TCSCS can improve sexual function.
Hypothesis 3B: Long-term TCSCS will improve sexual function in male and female participants, with an emphasis on overall sex life satisfaction. These improvements will be greater in individuals who complete intensive long term TCSCS compared to individuals who complete moderate long term TCSCS.
Outcome 3B: Mean improvement in satisfaction with participant's overall sexual life.

Objective 3C) To assess persisting effects of long-term TCSCS on LUT, bowel and sexual function.
Hypothesis 3C): The longevity of the beneficial effects on LUT, bowel and sexual function, after cessation of TCSCS, will be greater in the group that received intense stimulation versus the group that received moderate stimulation.
Outcome 3C): Maintained improvements in urinary and faecal incontinence (AIM 3A) and sexual function (AIM 3B) at 6 and 12 weeks after the cessation of TCSCS.

Research design

This is a proof of concept study to demonstrate the clinical efficacy of real-time (short-term) TCSCS (proof-of-concept) to improve LUT, and bowel in adults with chronic SCI. This is also a pilot trial phase and a proof-of-concept study to test clinical long term efficacy of TCSCS to improve LUT, bowel and sexual function in adults with chronic SCI.

Statistical Plan

Statistical analysis on LUT, bowel and sexual function will be performed (n = 40 with SCI).

A sample size calculation was performed using G*power, version 3.0.10, with power set at 0.8 and α at 0.05. Data from participants that match our inclusion criteria (n = 5, level of lesion C5 – T10, motor-complete AIS, A or B) were used from a recent TCSCS study.[30] A minimum sample of 26 participants is required to detect a significant change in voiding efficiency [voided volume / (voided volume + post-void residual volume)] with short-term TCSCS (stim-off = 37.8 ± 40.6% vs. stim-on = 58.2 ± 39.4%, effect size = 0.510). Presently, there are no long-term TCSCS data with LUT and bowel outcomes, consistent with those proposed in this protocol, to facilitate a meaningful power calculation. Therefore, given this is a pilot trial study, and in order to account for drop out with long-term TCSCS, we aim to investigate 40 individuals with SCI (n = 40, C5 – T6).Short-term TCSCS effects on UDS parameters (AIM 2A) as well as ARM testing (AIM 2C) with or without different stimulation configurations will be assessed using a repeated measures non-parametric analysis of variance (ANOVA), i.e. Kruskal–Wallis test. Data from the repeatability portion of the study (AIM 2A) will be assessed within (same session) and between (different sessions) visits (2 and 3) using a repeated measures non-parametric ANOVA. TNFBM will also be assessed using a repeated measures non-parametric ANOVA.

Long-term TCSCS: Differences in continuous variables for questionnaire data (NBSS, NBD Score, I-QoL, IIEF-15 or FSFI) in AIM 3 will be assessed using a two-way repeated measures [baseline, immediately post long term TCSCS as well as post-interventional follow up at 6 and 12 weeks] ANOVA comparing changes between moderate and intensive TCSCS. Furthermore, UDS testing as well as 'faecal continence severity' (baseline and end of TCSCS 6-week period) will be assessed using Wilcoxon signed-rank tests within

groups. A P-value less than 0.05 will be considered statistically significant. A complete thematic analysis will be performed to collect contextual qualitative data from the sexual health interview.

Participants

Enrollment Goal

40 participants with SCI.

Target Population

Adults 18-65 with >1-year post injury traumatic SCI at or above the T6 spinal segment with documented lower urinary tract dysfunction. (n = 40).

Inclusion and Exclusion Criteria

Inclusion:

A participant with SCI must meet all of the following criteria in order to be eligible for inclusion:

1. Resident of British Columbia, Canada with active provincial medical services plan
2. Male or female, 18-65 years of age
3. Chronic traumatic SCI (non-progressive, with complete motor paralysis) at or above the T6 spinal segment.
4. >1-year post injury, at least 6 months from any spinal surgery.
5. Documented presence of bladder dysfunction
6. Documented presence of bowel or sexual dysfunction.
7. American Spinal Injury Association Impairment Scale (AIS) A, B.
8. Greater than or equal to antigravity strength in deltoids and biceps bilaterally
9. Hand function sufficient to perform Clean Intermittent Catheterization (CIC) or a committed caregiver to provide CIC for management of urinary bladder drainage.
10. Participants must have documented three days of bladder and bowel history prior to their baseline visit.
11. Willing and able to comply with all clinic visits and study-related procedures.
12. Able to understand and complete study-related questionnaires (must be able to understand and speak English or have access to an appropriate interpreter as judged by the investigator).
13. No painful musculoskeletal dysfunction, unhealed fracture, pressure sore, or active infection that may interfere with testing activities.
14. Medication dosage must be stable for period of 4 weeks prior to participation
15. Baclofen dose cannot exceed 60mg per day
16. Stable management of spinal cord related clinical issues (i.e., spasticity management).
17. Women of childbearing potential must not be intending to become pregnant, currently pregnant, or lactating. The following conditions apply:
    a) Women of childbearing potential must have a confirmed negative pregnancy test prior to the baseline visit.
    b) Women of childbearing potential must agree to use adequate contraception during the period of the trial. Effective contraception includes abstinence.

18. Must provide informed consent.

Exclusion

A participant who meets any of the following criteria will be ineligible to participate in Study:

1. Presence of severe acute medical issue that in the investigator's judgement would adversely affect the participant's participation in the study. Examples include, but are not limited to clinically significant renal or hepatic disease; acute urinary tract infections; pressure sores; active heterotopic ossification; newly changed antidepressant medications [tricyclics]; or unstable diabetes. The following conditions apply:
   a. Moderate and severe forms of renal dysfunctions (eGFR below 60 ml/min)
   b. Clinically significant abnormal laboratory tests (ALT; Alkaline Phosphatase; Bilirubin [total]; GGT) as judged by the investigator.
2. Recent treatment with OnabotulinumtoxinA into the detrusor muscle (within 6 months of the baseline visit)
3. Persons requiring ventilator support
4. Clinically significant depression or ongoing drug abuse
5. Use of any medication or treatment that in the opinion of the investigator indicates that it is not in the best interest of the participant to participate in this study.
6. Intrathecal baclofen pump
7. Oral baclofen dose >60mg
8. Cardiovascular, respiratory, bladder, or renal disease unrelated to SCI or presence of hydronephrosis or presence of obstructive renal stones.
9. Any implanted metal in trunk or spinal cord under the sites of application of electrodes (between anode and cathode).
10. Any active implanted active implanted medical device, including, but not limited to neurostimulators, cochlear implants, pacemakers, implantable defibrillators, or drug delivery pumps.
11. Severe anemia (Hgb<8 g/dl) or hypovolemia
12. Participant is a member of the investigational team or his /her immediate family.
13. Participant has undergone electrode implantation surgery.
14. Participant with swollen, infected, and inflamed areas or open wounds on the area of stimulation

Overview

Eligible participants with SCI (n = 40) who can commit to 6 visits, over 4-6 weeks, will complete for the short-term effects of TCSCS. Participants with SCI (n = 40) who can commit to the full 25-week timeline will complete and then progress to complete for the long-term effects of TCSCS. Eligible participants, enrolled into will attend 5 visits at ICORD , 1 visit at St. Paul's hospital. Eligible participants will attend 37 visits at ICORD, 1 visit at St. Paul's Hospital, and 2 visits by phone. With the exception of ARM (conducted at Gastroenterology Clinic, St. Paul's hospital, Vancouver), all experiments will be performed at the International Collaboration on Repair Discoveries (ICORD), Blusson Spinal Cord Centre (BSCC), UBC. TPlease see the Schedule of Events in section 9 of the application.

Visit 0 Pre-Screening

Individuals who respond to study advertisements in BCSCI journal, ICORD website, or GF Strong bulletin board will be contacted by a clinical coordinator and will be pre-screened for eligibility (virtually or in

person) based on the study inclusion and exclusion criteria and the potential participant's self-report and/or medical records.

Based on this pre-screening, potential participants will be provided the study informed consent form via email, and will be given at least 24 hours to review the form and ask any potential questions and make their decision about potential participation in the study.

Visit 1 Screening – Weeks 1-2:

Approximately 1.5 hours

Individuals who agree to participate in the study will be invited for a screening assessment to confirm study eligibility. After the individual has provided informed consent, he/she will be assigned a unique study number and the information listed below will be collected. All eligible participants will undergo detailed neurological examinations in order to establish severity of SCI.

Screening Assessments:

- Inclusion/Exclusion Criteria confirmation
- Demographics
- Medical history and history of injury
- Concomitant medication use
- Weight and height
- Previous allergies and adverse events to medications
- Renal ultrasound will be included from annual evaluation of patients
- Pregnancy screening will be performed
- Depression screening - Patient Health Questionnaire-9 (PHQ-9) (please see Descriptions of Assessments for details)
- Autonomic dysfunction in Spinal Cord Injury (ADFSCI) questionnaire
- International Standards for Neurological Classification of Spinal Cord Injury (ISNCSCI) exam

Visit 2: Baseline Assessments – Weeks 3-4:

Approximately 1 hour.

All eligible participants will undergo baseline evaluation for severity of bladder, bowel, sexual and cardiovascular dysfunctions using the validated questionnaires listed below. Participants will also undergo baseline 24 hours ambulatory evaluation of cardiovascular functions. This test will be specifically scheduled on the day of bowel routine with detailed documentation of time of bowel routine and each of the bladder catheterisations.

Low Urinary tract assessments

- Neurogenic Bladder Symptom Score (NBSS)
- The Incontinence-QoL (I-QoL)

Bowel assessments

- Neurogenic bowel dysfunction score (NBD Score)
- The modified Wexner fecal incontinence score (WIS)
- Time Needed for Bowel Movement (TNFBM), at home

Sexual health assessments

- The International Index of Erectile Function (IIEF-15) (male participants)
- The Female Sexual Function Index (FSFI) (female participants)
- Pregnancy test for female participants.

Cardiovascular assessments

- 24 hrs BP monitoring (on day of bowel routine):
- 24 hrs diary of activities

Visit 3 /Baseline EMG Mapping of Spinal Cord Segments with TCSCS / Week 3-4

Approximately 2 hours.

Participants will undergo baseline mapping using surface EMG (Delsys Inc., Natick, USA) and concentric needle EMG as per an established protocol to record muscle activation during transcutaneous spinal cord stimulation (mapping) (TESCoN, SpineX Inc., CA, USA) at different spinal cord levels and different intensity. Please see subsections entitled TCSCS, Surface EMG and Concentric Needle EMG in Descriptions of Assessments section for details.

Visits 4,5,6: Urodynamics and Anorectal Manometry – Weeks 4-6:

Approximately 2.5 hours per visit.

Following Visits 1 (Screening) Visit 2 (baseline assessments) and Visit 3 (mapping visit) using a randomized counter-balanced approach, all individuals will be allocated into two distinct pathways:

- Pathway #1: individuals will start with Urodynamics, (UDS 1, Visit 4), followed by a second session of Urodynamics (UDS 2, Visit 5), and ARM (Visit 6).
- Pathway#2: individuals will start with ARM (Visit 4), followed by Urodynamics (UDS 1, Visit 5), and a second session of Urodynamics (UDS 2, Visit 6)).

TCSCS and Cardiovascular monitoring will be performed during UDS and ARM. Please see Description of Assessments for details of Urodynamics, ARM, TCSCS and Cardiovascular monitoring.

Visits 7 – 18 or 36: Long Term TCSCS – Weeks 7-12:

Approximately 90 minutes per visit.

Participants will come to ICORD for long term TCSCS, either 2 days/week (moderate) or 5 days/week (intense) for 6 weeks for a total of either 12 sessions or 30 sessions. Please see Description of Assessments for the details of an individual session.

During the 6 weeks of long term TCSCS, TNFBM will be measured during participants' conventional bowel routines at home. Participants will be asked to record the time from 'suppository insertion' to 'when bowel evacuation is completed' once per week for the bowel movement completed mid-week during each week of the 6 week TCSCS pathway.

24 ABPM will be performed mid-way through the 6 weeks and during the final week of the 6 weeks of treatment in combination with the participants' bowel routine.

Visit 19-20 OR 37 – 38: Immediate Post-TCSCS Assessments – Week : 13

Visit 19 or 37 (approximately 3 hours):
After the 6 weeks of TCSCS, all participants will complete 1 visit at ICORD including UDS assessments and a SCI severity assessment (ISNCSCI). Please see description of Assessments for details.

Visit 20 or 38 (approximately 1 hour):
A series of questionnaires on low urinary tract function, bowel function, cardiovascular function and sexual health will be conducted by telephone or video conference call (considering the present COVID-19 pandemic environment). The questionnaires are listed below and details can be found in the Description of Assessments Section.

Post-TCSCS Questionnaires

Low Urinary tract assessment

- Neurogenic Bladder Symptom Score (NBSS)
- The Incontinence-QoL (I-QoL)

Bowel assessment

- Neurogenic bowel dysfunction score (NBD Score)
- The modified Wexner fecal incontinence score (WIS)
- Time Needed for Bowel Movement (TNFBM), completed at home

Sexual health assessments

- The International Index of Erectile Function (IIEF-15) (male participants)
- The Female Sexual Function Index (FSFI) (female participants)

Cardiovascular assessments

- Autonomic dysfunctions in SCI (ADFSCI) questionnaire: Comprises of 21 questions, covering three domains, including general, medications, and autonomic dysreflexia symptoms and severity

Visit 21-22 or 39 - 40 / Follow-Up Visits at 6 and 12 weeks after completion of stimulation protocol/Week 19 and Week 25

These visits will take place over the telephone to evaluate existing and new adverse events and concurrent treatments. During each of these follow-up phone Visits (39 and 40) at 6 and 12 weeks post completion of long term TCSCS, a series of questionnaires on low urinary tract function, bowel function, cardiovascular function and sexual health will be conducted by telephone or video conference call (considering the present COVID-19 pandemic environment). The questionnaires are listed below and details can be found in the Description of Assessments Section.

During visit 40, a sexual health clinician, with experience providing clinical care to individuals with SCI, will also conduct a semi-structured qualitative interview. Please see Description of Assessments for details.

Visit 21/39 will take approximately 1 hour and visit 22/40 will take approximately 1.5 hours.

Low Urinary tract assessment

- Neurogenic Bladder Symptom Score (NBSS)
- The Incontinence-QoL (I-QoL)

Bowel assessment

- Neurogenic bowel dysfunction score (NBD Score)
- The modified Wexner fecal incontinence score (WIS)
- Time Needed for Bowel Movement (TNFBM), completed at home

Sexual health assessments

- The International Index of Erectile Function (IIEF-15) (male participants)
- The Female Sexual Function Index (FSFI) (female participants)

Cardiovascular assessments

- Autonomic dysfunctions in SCI (ADFSCI) questionnaire

Descriptions of Assessments

Physical examinations

SCI severity assessment:

- *International Standards for Neurological Classification of Spinal Cord Injury (ISNCSCI) examination:*
  Neurological evaluations of participants with SCI will be performed using the ISNCSCI exam. The level and severity of damage to motor and sensory pathways will be determined by a trained physician using the standard ISNCSCI examination (2019 revision).[31] In brief, this examination includes the assessment of motor function of key muscles in the upper and lower extremities using an established scale (muscle power graded as 0-5; for a total of 20 muscles in the four limbs) and sensory evaluation to light touch and pin prick in 28 dermatomes of the body. Sensory scores for each dermatome are assigned as 0=absent, 1=abnormal, and 2=normal. With this coding system, participants who perceive a pinprick as minimally sharp touch are assigned the same score as those who perceive it as almost normal. Perception of light touch stimuli is graded similarly. An examination of anal tone and sensation will be also conducted. The PI, Dr. Krassioukov and his Co-Investigators, have the necessary clinical training to perform this evaluation, and will be responsible for this part of the study. This evaluation will be performed by either Dr. Krassioukov (MD, PhD) or Dr. Berger (MD, PhD), who are both Physical Medicine and Rehabilitation physicians.

Procedures

TCSCS

TCSCS will be delivered using a non-invasive central nervous system stimulator (TESCoN, SpineX Inc., CA, USA). A self adhesive round electrode placed on the skin between spinous processes at the midline over the vertebral column will function as a cathode, and two rectangular self-adhesive electrodes located

symmetrically on the skin over the iliac crests as anodes. To activate the spinal circuits known to be involved in LUT and bowel control (AIM 1), TCSCS will be delivered individually between spinous processes T10-11, T11-12, T12-L1, and L1-L2 (conus medullaris). Stimulation will be applied at various frequencies ranging between 1Hz and 90Hz. Current amplitude will start at 10mA and will be increased in incremental steps until tolerable or until responses plateau. Specific areas for electrode placement will be examined and prepared to reduce skin impedance. A temperature probe will be positioned adjacent to the cathode in order to monitor changes in skin temperature to prevent potential skin damage. TCSCS will be delivered under the supervision of either Dr. Krassioukov or Dr. Berger, who are both Physical Medicine and Rehabilitation physicians.

Surface EMG

EMG signals will be recorded using a commercially available Delsys system and custom LabVIEW software (LabVIEW, National Instruments, USA). EMG data will be analyzed offline using custom MATLAB routines (MathWorks, Natick, USA). EMG signals will be sampled at 10KHz, bandpass filtered (5Hz-5KHz) and rectified for analysis. Potential motor targets and muscles we will record from include: T10-12= lower abdominal [rectus abdominis], L1-2= hip flexor [rectus femoris], L3= knee extensors [vastus medialis and lateralis], L4= ankle dorsiflexors (tibialis anterior), L5= long toe extensor [hallucis longus], S1= ankle plantar flexors [gastrocnemius], S2-3= knee flexion (hamstring), S2-S4 perineum (only control participants). Parameters of interest will be rectified surface EMG, time-locked to the electrical stimulation in 300 us bins. Motor evoked potential latency and amplitude will be recorded if present. Surface EMG will be recorded under the supervision of either Dr. Krassioukov or Dr. Berger, who are both Physical Medicine and Rehabilitation physicians.

Concentric Needle EMG

Monopolar concentric needle EMG will be used to evaluate for evidence of denervation and for more sensitive evaluation of voluntary motor activity. Concentric needle EMG will only be used to examine the external anal sphincter muscle, due to the very small size of muscle.

A standard clinical monopolar needle electrode will be inserted into the external anal sphincter muscle, with additional reference surface electrodes positioned adjacent to the site of needle insertion. A ground electrode will be positioned over a nearby bony prominence (Anterior Superior Iliac Crest). EMG signals will be recorded using a commercially available pre-amplifier (1902, Cambridge Electronic Design, Cambridge UK), analog-to-digital converter (1401, Cambridge Electronic Design, Cambridge UK) and recorded in commercially available data acquisition software (Spike2, Cambridge Electronic Design, Cambridge UK). EMG signals will be sampled at 20KHz, bandpass filtered (10Hz-10KHz) and analysed offline. Concentric needle EMG will be performed by Dr. Berger, who is a Physical Medicine and Rehabilitation physician.

Urodynamics

Participants will undergo standard clinical procedures for UDS, including cystometry with water at 21°C and a filling rate of < 30 mL per minute through a 6F double lumen catheter with the participants in the supine position. Abdominal pressure will be measured with a 10F intrarectal balloon catheter. Pelvic floor electromyography will be recorded during cystometry with a surface electrode placed on the

perineum. Filling will be stopped when the participants report a sensation of fullness, at the moment of urine leakage, if blood pressure reaches levels of 180/110 mmHg, or per the request of the patient.

Following the standard clinical assessment UDS we will perform UDS with TCSCS. To assess the efficacy of short-term TCSCS' on LUT function, we will first prefill the bladder (i.e. 50-75% of cystometric capacity from the first UDS) without eliciting detrusor overactivity or autonomic dysreflexia. Then, TCSCS (as described above) will be delivered for 60 s each interspersed with 30 s rest (i.e. no stim) to optimize the configuration for each individual. Stimulation frequency and location will be guided by the results of the Baseline EMG Mapping of Spinal Cord Segments performed during visit 3.

TCSCS will be stopped when the participants report a sensation of fullness, at the moment of urine leakage, if blood pressure reaches levels of 180/110 mmHg, or per the request of the patient.

Urodynamics will be performed by a trained clinician (MD or RN) under the supervision of Dr. Kavanagh (MD), who is a urologist.

Cardiovascular monitoring

Cardiovascular monitoring will be utilised as a safety measure to detect and record potential ACVEs. such as autonomic dysreflexia, during UDS and ARM (with and without TCSCS). BP will be measured continuously (beat-by-beat) via finger photoplethysmography corrected to brachial pressure and heart rate, measured via one-lead electrocardiogram. An attending physician will monitor for potential development of episodes of autonomic dysreflexia, which is defined according to the International Standards to document remaining Autonomic Function after SCI as an increase in systolic blood pressure ≥20mmHg from baseline. Autonomic dysreflexia will be resolved with the implementation of an established clinical management protocol in accordance with the Consortium of Spinal Cord Medicine Clinical practice guidelines (Clinical Practice guidelines provide recommendations for healthcare specialists based on evidence-based research conducted by the Consortium for Spinal Cord Medicine).

ARM

This is a well-established methodology that provides a direct assessment of anal sphincter pressure and anorectal coordination during simulated defaecation. ARM is widely available, easy to perform and well accepted by participants.[32] ARM will be repeated with TCSCS according to the protocol for TCSCS application as described above. Stimulation frequency, location and intensity during ARM will be guided by the results of the Baseline EMG Mapping of Spinal Cord Segments performed during visit 3 and by our ability to activate S2-S4 innervated muscles (spinal cord segments crucial for anorectal sphincter control).

24 hrs BP monitoring (on day of bowel routine):

A 24-hour ABPM will be performed using the Meditech Card(X)plore (Meditech Ltd., Budapest, Hungary), a well-established clinical protocol[33] on a day the participant completes a bowel routine.. Compared to a single one-time clinic visit, 24-hour ABPM has been found to provide a highly effective and comprehensive assessment of true blood pressure and heart rate.[33] Briefly, a standard adult-sized cuff for an arm circumference of 24-32 cm will be fixed on the non-dominant arm. A mercury sphygmomanometer comes attached to the monitor and recordings will be taken every 15 minutes between 0700-2300, which represents the daytime period, and then every hour from 2300-0700, which represents the night-time period. Daytime and night-time values will be determined from the average of all measurements during each time period. All participants will be asked to complete an activity log to

indicate the time before and after each CIC, transfers, time they transferred into supine position to sleep then seated when they woke-up, or any other times they felt AD occurring. Additionally, all participants will be instructed to manually record a BP measurement during each event by pressing the button ‘1’ followed by the button ‘2’ located on the front of the ABPM device. Participants will be instructed to maintain their routine activities while avoiding exercise and excessive physical exertion. Participants will be also requested to avoid scheduling their regimented bowel routine (as a common AD trigger) on the same day of wearing the ABPM. Prior to starting 24-hour ABPM, three consecutive baseline measurements (separated by one minute) will be recorded with the participant seated in their own wheelchair, following five minutes of rest. The average of three measurements blood pressure and heart rate) in the seated position will be used as the baseline from which the presence or absence of AD bouts during the daytime 24-hour ABPM period will be determined. Any measurement that had a systolic blood pressure ≥20 mm Hg above the average seated baseline value will be documented as a bout of AD, and the corresponding cause was obtained from the activity log. AD severity will be calculated by subtracting the highest recorded systolic blood pressure from the respective baseline. Calculation of AD during the night required another baseline BP due to change in position from seated to supine and due to a physiological nocturnal dip that is found absent in those with motor-complete tetraplegia. A nocturnal dip is described as a physiological circadian blood pressure pattern where blood pressure is reduced by 10% during sleep.[33] Participants will be given a diary to log their activities while wearing the 24 hour blood pressure monitor.

Long Term TCSCS Visits

Each session will last approximately 90 minutes. TCSCS will be delivered using a non-invasive central nervous system stimulator (TESCoN, SpineX Inc., CA, USA). A self adhesive round electrode placed on the skin between spinous processes at the midline over the vertebral column will function as a cathode, and two rectangular self-adhesive electrodes located symmetrically on the skin over the iliac crests as anodes. Specific areas for electrode placement will be examined and prepared to reduce skin impedance. A temperature probe will be positioned adjacent to the cathode in order to monitor changes in skin temperature to prevent potential skin damage. Blood pressure and heart rate will be measured once every minute throughout the. TCSCS will be delivered under the supervision of either Dr. Krassioukov or Dr. Berger, who are both Physical Medicine and Rehabilitation physicians.

Validated questionnaires

Depression screening - Patient Health Questionnaire-9 (PHQ-9)

This scale is a brief, 9-item self-report screening tool that meets the criteria for good diagnostic accuracy for assessing depressive disorders during inpatient SCI rehabilitation.[34] A comprehensive systematic review performed by the Spinal Cord Injury Research Evidence (SCIRE) Team concluded that given the adequate to excellent reliability and validity of this measure there is no requirement to develop an SCI-specific instrument.[35]

Autonomic dysfunction in Spinal Cord Injury (ADFSCI) questionnaire assesses self-reported frequency and severity of autonomic related symptoms. The AD part of the questionnaire includes 10 items, each using a 5-point scale to score the frequency and severity of hypertensive symptoms, such as headache, goose bumps, confusion, etc., under different circumstances.

Low Urinary tract assessments

- Neurogenic Bladder Symptom Score (NBSS): comprises 23 questions covering 3 domains, including incontinence (primary outcome, AIM 3A), storage & voiding, and specific consequences, as well as one question on QoL.[36]
- The Incontinence-QoL (I-QoL): comprises 10 questions covering 3 domains, including avoidance and limiting behaviour, psychosocial impacts, and social embarrassment, which will be summarised as a total score. All scores, for each domain and a total, will be transformed into a continuous scale value.[37]

Bowel assessments

- Neurogenic bowel dysfunction score (NBD Score) The NBD score is a measure of both constipation and fecal incontinence and was developed for and validated in the SCI population[38]. This questionnaire comprises 10 questions focusing on defecation (i.e., frequency, duration, and clinical symptoms), constipation (i.e., use of aiding medication and digital stimulation), fecal incontinence (i.e., frequency, aiding medication, and flatus; and peri-anal skin problems. The consequential NBD score relates to four different neurogenic bowel dysfunction severity levels[39]
- The modified Wexner fecal incontinence score (WIS): The WIS scale includes items that take into account type, frequency and extent to which fecal incontinence alters an individual's life, use of anti-diarrhea medications and the ability to defer defecation for 15 min.[40]
- Time Needed for Bowel Movement: Participants will be asked to record the time from 'suppository insertion' to 'when bowel evacuation is completed', during their regular bowel movement at home.

Sexual health assessments

- The International Index of Erectile Function (IIEF-15) (male participants): comprises of 15 questions covering five domains, including erectile function, orgasmic function, intercourse satisfaction, and overall satisfaction.[41]
- The Female Sexual Function Index (FSFI) (female participants): comprises of 19 questions covering six domains, including desire, arousal, lubrication, orgasm, satisfaction and pain.[42]
- Pregnancy test for female participants: A pregnancy test will be required for any female participants of child bearing potential to ensure the participant is not pregnant.

Cardiovascular assessments

- Autonomic dysfunctions in SCI (ADFSCI) questionnaire: Comprises of 21 questions, covering three domains, including general, medications, and autonomic dysreflexia symptoms and severity
- 24 hrs diary of activities: Participants are given a diary to log their activities while wearing the 24 hour blood pressure monitor.

Semi-Structured Qualitative Interview

Participants will be interviewed in person by a sexual medicine clinician at the Blusson Spinal Cord Injury Centre to ascertain qualitative information on sexual functioning. These interviews will be digitally recorded, transcribed verbatim and anonymized on completion. The interview schedule will include questions pertaining to the following: participants sexual history and current relationship status, level of

sexual drive, ability to orgasm, barriers to sexual activity, self-esteem, the use of ergogenic aids, and participants' perceptions of the role of TCSCS on sexual functioning. Female participants will be asked about lubrication quality, and male participants will be asked about erection quality (with and without pharmaceutical or prosthetic assistance), and their ability to ejaculate. Participants will be advised during the consent process that the clinician will be acting as a researcher during this interview and is therefore prohibited from providing clinical care or direct medical advice during the study. A complete thematic analysis will be performed using NVivo software (Version 9.0, QSR, Southport, UK). The expected duration of the interview will be approximately 30 – 45 minutes.

Known Study Risks/Harms

- Through the period of study, any adverse event will be recorded using the adverse event report form in section 9 of the application. If an adverse event occurs during the testing session will be immediately stopped. In addition, the participant's primary care provider and UBC CREB will be notified as necessary if serious adverse events (SAEs) occur. In addition, the participant's primary care provider, UBC CREB, and the Data Monitoring Committee (DMC) will be notified as necessary if serious adverse events (SAEs) occur. A Data and Safety Monitoring Committee will comprise of 3 individuals external and independent to the study to review the adverse event reports and stop the study if the safety data are of sufficient concern. Any adverse effects will be reported to these individuals responsible for Data and Safety Monitoring Committee, as well as to the appropriate UBC ethics boards by the study coordinator.

- The urodynamics and ARM procedure will necessitate that the participant expose their genitals in the presence of the Principal Investigator or study health care professionals. Every effort will be made to ensure their comfort and privacy during the procedure as long as safety is assured. For the urodynamic assessment, once the catheter is implanted, the gown will be placed to cover their genitals. Due to the risk of high blood pressure, participants will not be left unattended during urodynamic assessments.

- Participants may experience stress as a result of answering questions related to symptoms of depression, bowel and bladder function, and sexual functioning/satisfaction. The depression, bowel and bladder function questionnaires used in this study are commonly used clinical screening tools. The sexual functioning/satisfaction questionnaires were carefully chosen as they have previously been used in individuals with SCI. These questionnaires will be delivered in a private setting, by a single sexual medicine clinician, with considerable experience delivering these assessment tools and discussing such sensitive topics with male patients with SCI. Participants will also be provided with the Fraser Health Crisis line in the informed consent document if they require immediate, free and confidential emotional support.

- Participants will be advised not to take Viagra, Clans or Levitra for 4 days before the urodynamic tests as this could cause a drop in blood pressure requiring medical attention.

- With TCSCS we do not expect adverse events as our study will only utilize parameters and electrodes approved in previous studies.[43,44] Stimulation could potentially irritate the skin, so we will monitor skin temperature for potential irritation. TCSCS elicit AD, however so far no AD/OH is observed in TCSCS studies. Furthermore, cessation in stimulation immediately reduces blood pressure, and we will be monitoring blood pressure closely during procedures. In case of adverse

events, the participant's primary physician would be notified as needed. Participants will be continuously monitored for any signs of risks or discomfort. As mentioned, we will also continuously monitor cardiovascular signals such as blood pressure and heart rate. For TCSCS, we will additionally measure skin temperature.

- In this study, participants will be asked to undergo UDS, a clinically approved procedure for examination of urinary bladder function in participants with injury to the central or periphery nervous system. This procedure is typically recommended to be performed on a biannual basis in participants with multiple sclerosis, stroke, and SCI, in order to establish urine bladder function. It is known that within the SCI population, UDS commonly elicits episodes of AD in participants with injury at the T6 spinal segment and above (an inclusion criteria for the present study). Clinically, UDS is an important component of bladder function evaluation following SCI.[45] Risk management in this study involves having trained personnel performing these studies in a clinical facility with protocols in place to manage iatrogenic/artificially induced episodes of AD. All these criteria are in place at the site of the Principal Investigator, located at the ICORD/Blusson Spinal Cord Injury Centre at the Vancouver General Hospital.
- Concentric needle EMG is a common clinical procedure, performed in a variety of clinical settings worldwide. The risks of EMG are very small and include small risk of bleeding, bruising and mild discomfort that can last up to 24 hours. EMG is very well tolerated and the risks will be minimized by localizing the target muscles using well-established surface anatomical landmarks in order to avoid placement in proximity to blood vessels or nerves. In the event of discomfort during the procedure, this will be minimized simply by moving the needle to a slightly different position.

Managing episodes of AD:

The present concept of management of AD following SCI consists of preventative and treatment strategies.

Prevention

Adherence to the bladder protocol and prevention of urinary tract infections is highly recommended as up to 90% of episodes of AD are related to the urogenital tract. Adherence to proper bladder management and regular bowel protocols is also highly recommended in order to prevent most common triggers of AD. Skin checkup and toenail care are crucial for participants with SCI as skin issues are among frequent triggers of AD episodes.

Treatment

Non-pharmacological components for management of AD episodes include the following:

1. When an episode of AD is confirmed, the first recommended step is to sit the participant upright or elevate the head of the bed in an attempt to induce an orthostatic decline in blood pressure. Additionally, loosen or remove all restrictive clothing and devices that could be triggers of painful stimuli (tight belt, shoe lace, orthotics on the leg, etc).
    - After this step measure blood pressure - this step could be sufficient to eliminate the episode of AD. If blood pressure is still elevated go to step 2.
2. Check the urinary bladder. If the catheter is inserted or kinked; establish urine outflow.

- After this step measure the blood pressure again and if the blood pressure is still elevated go to step 3.

3. Check for possible fecal impaction (use lidocaine gel to decrease irritation).
    - After this step measure the blood pressure again and if the blood pressure is normalized and symptoms subside, AD is successfully managed.
    - However, if arterial blood pressure is still elevated and it is above 150 mmHg the next step in management is initiation of pharmacological agents.

Based on literature and recommendations, the present pharmacological arsenal for management of AD episodes in North America includes the following:

1. Nifedipine (5mg capsule)
2. Captopril (25mg capsule)
3. Nitrates (1-2 inches applied to non-hairy part of chest)

These recommendations were developed and based on the consensus of specialists who were involved in development of PVA guidelines for management of AD following SCI.

The costs of all tests, examinations, and medical care required as a part of this study will be provided at no cost to the study subject. The subject and others may benefit from the overall conclusions to be drawn from the results of this study. Participation in this study could provide further data on the impact of TCSCS on lower urinary tract, bowel and sexual function following SCI.

Ethical and Regulatory Considerations

Good Clinical Practice (GCP) Statement

The sponsor investigator recognizes that it is his responsibility to ensure that this clinical study will be conducted in accordance with the ethical principles that have their origin in the Declaration of Helsinki, and that are consistent with the International Conference of Harmonization (ICH) guidelines for GCP and applicable regulatory requirements.

Informed Consent

The principles of informed consent described in the ICH Guidelines for GCP will be followed when enrolling participants into this study. The Informed Consent Form (ICF) used in this study must be reviewed and approved by UBC Clinical Research Ethics Board (UBC CREB).

Written informed consent will be obtained from each participant prior to their participation in any study procedure. The original ICF will be retained in the participant’s study record and a copy of the signed ICF will be given to the participant. The same protocol applies to any amendments to the original consent; all versions of signed consents will be kept for the Health Canada regulated period of 25 years.

Participant Confidentiality and Data Protection

The investigator will take all appropriate measures to ensure that the anonymity of each study participant will be maintained. Participants will be assigned study specific numbers and study specific initials that are not based on any personal identifiers. The identification key linking participants to their study identifiers will be kept in strict confidence and access restricted to appropriate study personnel.

The investigator will take all appropriate measures to safeguard and prevent access to this information by any unauthorized third parties. However, research records and health or other source records identifying you may be inspected in the presence of the Investigator or his or her designate, by representatives of Health Canada, and the UBC Clinical Research Ethics Board for the purpose of monitoring the research. No information or records that disclose participants identity will be published without their consent, nor will any information or records that disclose participant identity be removed or released without their consent unless required by law.

Remuneration of Participants

Participants with SCI will receive $500 CAD upon study completion of the study to offset the cost of transit, or other transportation costs involved with study visits.

Control participants will receive a $50 gift card upon study completion.

Bibliography

1. Hou, S. & Rabchevsky, A. G. Autonomic consequences of spinal cord injury. *Compr. Physiol.* **4,** 1419–1453 (2014).
2. Krassioukov, A. Autonomic function following cervical spinal cord injury. *Respir. Physiol. Neurobiol.* **169,** 157–164 (2009).
3. Anderson, K. D. Targeting recovery: priorities of the spinal cord-injured population. *J. Neurotrauma* **21,** 1371–1383 (2004).
4. Wheeler, T. L. *et al.* Translating promising strategies for bowel and bladder management in spinal cord injury. *Exp. Neurol.* **306,** 169–176 (2018).
5. Hammell, K. R. W. Spinal cord injury rehabilitation research: patient priorities, current deficiencies and potential directions. *Disabil. Rehabil.* **32,** 1209–1218 (2010).
6. Teasell, R. W., Arnold, J. M., Krassioukov, A. & Delaney, G. A. Cardiovascular consequences of loss of supraspinal control of the sympathetic nervous system after spinal cord injury. *Arch. Phys. Med. Rehabil.* **81,** 506–516 (2000).
7. Lee, A. H. X. *et al.* Alarming blood pressure changes during routine bladder emptying in a woman with cervical spinal cord injury. *Spinal Cord Series and Cases* **3,** 17101 (2017).
8. Liu, N., Zhou, M. W., Biering-Sørensen, F. & Krassioukov, A. V. Cardiovascular response during urodynamics in individuals with spinal cord injury. *Spinal Cord* **55,** 279–284 (2017).
9. Faaborg, P. M. *et al.* Autonomic dysreflexia during bowel evacuation procedures and bladder filling in subjects with spinal cord injury. *Spinal Cord* **52,** 494–498 (2014).
10. Panicker, J. N., Fowler, C. J. & Kessler, T. M. Lower urinary tract dysfunction in the neurological patient: clinical assessment and management. *Lancet Neurol.* **14,** 720–732 (2015).
11. Fowler, C. J., Griffiths, D. & de Groat, W. C. The neural control of micturition. *Nat. Rev. Neurosci.* **9,** 453–466 (2008).
12. de Groat, W. C. & Yoshimura, N. Mechanisms underlying the recovery of lower urinary tract function following spinal cord injury. *Prog. Brain Res.* **152,** 59–84 (2006).
13. Weld, K. J. & Dmochowski, R. R. Association of level of injury and bladder behavior in patients with post-traumatic spinal cord injury. *Urology* **55,** 490–494 (2000).
14. Burns, A. S. *et al.* Phenomenological study of neurogenic bowel from the perspective of individuals living with spinal cord injury. *Arch. Phys. Med. Rehabil.* **96,** 49–55 (2015).
15. Krassioukov, A., Eng, J. J., Claxton, G., Sakakibara, B. M. & Shum, S. Neurogenic bowel management after spinal cord injury: a systematic review of the evidence. *Spinal Cord* **48,** 718–733 (2010).
16. Furness, J. B., Callaghan, B. P., Rivera, L. R. & Cho, H.-J. The enteric nervous system and gastrointestinal innervation: integrated local and central control. *Adv. Exp. Med. Biol.* **817,** 39–71 (2014).
17. Singh, A., Tetreault, L., Kalsi-Ryan, S., Nouri, A. & Fehlings, M. G. Global prevalence and incidence of traumatic spinal cord injury. *Clin Epidemiol* **6,** 309–331 (2014).
18. Coggrave, M., Norton, C. & Wilson-Barnett, J. Management of neurogenic bowel dysfunction in the community after spinal cord injury: a postal survey in the United Kingdom. *Spinal Cord* **47,** 323–30; quiz 331 (2009).
19. Glickman, S. & Kamm, M. A. Bowel dysfunction in spinal-cord-injury patients. *Lancet* **347,** 1651–1653 (1996).
20. Stone, J. M., Nino-Murcia, M., Wolfe, V. A. & Perkash, I. Chronic gastrointestinal problems in spinal cord injury patients: a prospective analysis. *Am. J. Gastroenterol.* **85,** 1114–1119 (1990).
21. Lynch, A. C., Antony, A., Dobbs, B. R. & Frizelle, F. A. Bowel dysfunction following spinal cord injury. *Spinal Cord* **39,** 193–203 (2001).

22. in *Spinal Cord Medicine* (eds. Kirshblum, S. & WV, L.) 411–435 (Springer Publishing, 2019).
23. Cobo Cuenca, A. I., Sampietro-Crespo, A., Virseda-Chamorro, M. & Martín-Espinosa, N. Psychological impact and sexual dysfunction in men with and without spinal cord injury. *J. Sex. Med.* **12,** 436–444 (2015).
24. Sramkova, T. *et al.* Women's sex life after spinal cord injury. *Sex. Med.* **5,** e255–e259 (2017).
25. Simpson, L. A., Eng, J. J., Hsieh, J. T. C., Wolfe, D. L. & Spinal Cord Injury Rehabilitation Evidence Scire Research Team. The health and life priorities of individuals with spinal cord injury: a systematic review. *J. Neurotrauma* **29,** 1548–1555 (2012).
26. New, P. W. Secondary conditions in a community sample of people with spinal cord damage. *J Spinal Cord Med* **39,** 665–670 (2016).
27. Reitz, A., Tobe, V., Knapp, P. A. & Schurch, B. Impact of spinal cord injury on sexual health and quality of life. *Int J Impot Res* **16,** 167–174 (2004).
28. Phillips, A. A., Elliott, S. L., Zheng, M. M. Z. & Krassioukov, A. V. Selective alpha adrenergic antagonist reduces severity of transient hypertension during sexual stimulation after spinal cord injury. *J. Neurotrauma* **32,** 392–396 (2015).
29. Wan, D. & Krassioukov, A. V. Life-threatening outcomes associated with autonomic dysreflexia: a clinical review. *J Spinal Cord Med* **37,** 2–10 (2014).
30. Gad, P. N., Kreydin, E., Zhong, H., Latack, K. & Edgerton, V. R. Non-invasive Neuromodulation of Spinal Cord Restores Lower Urinary Tract Function After Paralysis. *Front. Neurosci.* **12,** 432 (2018).
31. ASIA and ISCoS International Standards Committee. The 2019 revision of the International Standards for Neurological Classification of Spinal Cord Injury (ISNCSCI)-What's new? *Spinal Cord* **57,** 815–817 (2019).
32. Carrington, E. V. *et al.* Expert consensus document: Advances in the evaluation of anorectal function. *Nat. Rev. Gastroenterol. Hepatol.* **15,** 309–323 (2018).
33. Hubli, M. & Krassioukov, A. V. Ambulatory blood pressure monitoring in spinal cord injury: clinical practicability. *J. Neurotrauma* **31,** 789–797 (2014).
34. Bombardier, C. H. *et al.* Validity of the Patient Health Questionnaire-9 in assessing major depressive disorder during inpatient spinal cord injury rehabilitation. *Arch. Phys. Med. Rehabil.* **93,** 1838–1845 (2012).
35. Sakakibara, B. M., Miller, W. C., Orenczuk, S. G., Wolfe, D. L. & SCIRE Research Team. A systematic review of depression and anxiety measures used with individuals with spinal cord injury. *Spinal Cord* **47,** 841–851 (2009).
36. Welk, B. *et al.* The validity and reliability of the neurogenic bladder symptom score. *J. Urol.* **192,** 452–457 (2014).
37. Patrick, D. L. *et al.* Quality of life of women with urinary incontinence: further development of the incontinence quality of life instrument (I-QOL). *Urology* **53,** 71–76 (1999).
38. Krogh, K., Christensen, P., Sabroe, S. & Laurberg, S. Neurogenic bowel dysfunction score. *Spinal Cord* **44,** 625–631 (2006).
39. Alexander, M. S. *et al.* International standards to document remaining autonomic function after spinal cord injury. *Spinal Cord* **47,** 36–43 (2009).
40. Vaizey, C. J., Carapeti, E., Cahill, J. A. & Kamm, M. A. Prospective comparison of faecal incontinence grading systems. *Gut* **44,** 77–80 (1999).
41. Rosen, R. C. *et al.* The international index of erectile function (IIEF): a multidimensional scale for assessment of erectile dysfunction. *Urology* **49,** 822–830 (1997).
42. Rosen, R. *et al.* The Female Sexual Function Index (FSFI): a multidimensional self-report instrument for the assessment of female sexual function. *J Sex Marital Ther* **26,** 191–208 (2000).
43. Kreydin, E., Zhong, H., Latack, K., Ye, S., Edgerton, V.R. and Gad, P. Transcutaneous electrical spinal

cord neuromodulator (TESCoN) improves symptoms of overactive bladder. *Frontiers in systems neuroscience,* **14**, p.1 (2020).

44. Gad, P., Kreydin, E., Zhong, H. and Edgerton, V.R. May. Training the bladder how to void: A noninvasive spinal neuromodulation case study. *In 2021 10th International IEEE/EMBS Conference on Neural Engineering (NER)* (pp. 1010-1013). (2021).
45. Shin, J. C., Lee, Y., Yang, H. & Kim, D. H. Clinical significance of urodynamic study parameters in maintenance of renal function in spinal cord injury patients. *Ann Rehabil Med* **38,** 353–359 (2014).
